# The role of platelet to mean platelet volume ratio in the identification of adult-onset still’s disease from sepsis

**DOI:** 10.6061/clinics/2021/e2307

**Published:** 2021-04-07

**Authors:** Suohua Ge, Yongbin Ma, Mengxiao Xie, Tengfei Qiao, Jun Zhou

**Affiliations:** I Department of Neurology Laboratory, Jintan Hospital, Jiangsu University, Jintan, China; II Department of Laboratory Medicine, the First Affiliated Hospital of Nanjing Medical University, Nanjing, Jiangsu, China; III Department of Laboratory Medicine, Nanjing Lishui District Hospital of traditional Chinese medicine, Nanjing, Jiangsu, China

**Keywords:** Adult-Onset Still’s Disease, Sepsis, Platelet, Mean Platelet Volume, Identification

## Abstract

**OBJECTIVES:**

Inflammatory factors exert a significant role in the development of adult-onset Still’s disease (AOSD) and sepsis. Although platelet counts and platelet parameters have long served as indicators for inflammatory diseases, their role in the differential diagnosis between adult-onset stilĺs disease and sepsis remains unclear. We designed this retrospective study to explore whether the platelet to mean platelet volume (MPV) ratio (PMR) can help to distinguish AOSD from sepsis.

**METHODS:**

A total of 110 AOSD patients and 84 sepsis patients were enrolled in the study. Seventy-three AOSD patients and 56 sepsis patients between January 2010 and June 2017 were enrolled in the test cohort to analyze PMR values, which was then validated in the validation cohort (37 AOSD patients and 28 sepsis patients between June 2017 and December 2019).

**RESULTS:**

The values of PMR were significantly higher in AOSD patients than in sepsis patients (test cohort, validation cohort, and entire cohort), In the test cohort, logistic regression analysis showed that PMR was an independent risk factor of AOSD (odds ratios [OR]: 9.22, 95% confidence interval [CI] 2.15-39.46, *p=*0.003). Further receiver operating characteristic curve (ROC) analysis showed that the area under the ROC curve was 0.735 (95% CI 0.631-0.839, *p<*0.001) for PMR alone and 0.925 (95% CI 0.869-0.980, *p<*0.001) for the combination of PMR and serum ferritin. Consistently, the validation cohort exhibited analogous results.

**CONCLUSIONS:**

PMR could be used as a single indicator or a complementary indicator to distinguish AOSD from sepsis.

## INTRODUCTION

Adult-onset still’s disease (AOSD) is rare inflammatory arthritis characterized by the classic triad of fever, joint pain, and a distinctive salmon-colored bumpy rash, which was first proposed in the 1970s (1). Although increasing numbers of cases have been reported and certain inflammatory factors have been demonstrated to be associated with AOSD, the puzzling nature of AOSD is still far from being revealed (2,3). In particular, AOSD presents with a variety of clinical features resembling sepsis, making it difficult to diagnose. Despite the low incidence, AOSD patients still have both mental and physical pains from misdiagnosis and delayed treatment. Thus, it is important to enhance the diagnostic accuracy rate of AOSD, especially to distinguish it from sepsis, as soon as possible. Owing to the rare occurrence of AOSD and lack of disease-specific indexes, tumors, infections, and other inflammatory diseases are often given priority (4). Therefore, a specific indicator to AOSD is needed.

Ferritin and glycated ferritin are probably the most used biomarkers so far and have been widely assessed (5,6). However, high levels of ferritin could be indicators in different diseases such as tumors, infections, and sepsis. Glycated ferritin measurement is difficult to automate today. Until now, no ideal identity indicator can identify AOSD from sepsis.

Complete blood count test is a simple and common measurement, which is essential to check in both inpatients and outpatients. Platelet parameters, including plateletcrit (PCT), mean platelet volume (MPV), and platelet distribution width (PDW), have been extensively used in the diagnosis of disease, monitoring of treatment efficacy, prognosis, and disease recurrence (7-9). Regarding pediatric patients with infectious mononucleosis, the MPV to platelet (PLT) ratio was used as a biomarker to monitor abnormal liver function (10). However, there are no reports on its diagnostic value in distinguishing AOSD from sepsis. Therefore, we performed this retrospective study to evaluate whether the PLT to MPV (PMR) ratio can be used as an independent and/or accessory tool to distinguish AOSD patients from sepsis patients.

## MATERIAL AND METHODS

### Patients

This retrospective cohort study, conducted between January 2010 and December 2019, included newly diagnosed 110 AOSD patients [patients met the Yamaguchi criteria (4): meeting at least five criteria with two or more major criteria, no exclusion criteria]. The major criteria included: 1) fever >39°C for at least 1 week; 2) joint pain or arthritis that had lasted for ≥2 weeks; 3) typical skin rash; and 4) leukocytosis ≥10×10^9^/L with at least 80% granulocytes. The minor criteria included: 1) sore throat; 2) splenomegaly/lymphadenopathy; 3) absence of RF or antinuclear antibodies; and 4) impaired liver function and 89 sepsis inpatients [meeting two or more of the following conditions: (1) temperature >38°C or <36°C; (2) heart rate >90 beats/min; (3) respiratory rate >24 breaths/min or partial pressure of carbon dioxide <32 mm Hg; (4) white blood cell (WBC) count >12×10^9^/L, <4×10^9^/L, or >10% immature forms] (11) at the First Affiliated Hospital of Nanjing Medical University. Confounding factors including age (<16 years), cancers, receiving chemotherapy, glucocorticoids, and other autoimmune diseases or treatments that were likely to affect hematological parameters were excluded. The included patients were divided into two cohorts: the test cohort included 73 AOSD patients and 56 sepsis patients between January 2010 and June 2017; the validation cohort included 37 AOSD patients and 28 sepsis patients between June 2017 and December 2019. The study was confirmed by the Ethics Committee of the First Affiliated Hospital of Nanjing Medical University and was in line with the Declaration of Helsinki.

### Data collection

On admission before any treatment, the patients’ blood samples were collected within 2h for the subsequent measurement by Sysmex XE 2100 analyzers (Sysmex, Hyogo, Japan) (complete blood count), BN II nephelometer (Dade Behring, Marburg, Germany) (C-reactive protein [CRP]) and Unicel DXI 800 (Beckman Coulter, Brea, CA, America) (ferritin). The demographics, clinical features, and laboratory values of patients were downloaded from the medical database.

### Statistical analyses

All data analyses were conducted on SPSS version 21 software (SPSS Inc., Chicago, IL, USA) and data are presented as means±standard deviations, medians (interquartile ranges), or frequencies and percentages. The Shapiro-Wilk test was employed to check the distribution of the variables. Spearman/Pearson correlation, one-way analyses of variance, and Mann-Whitney *U*-test were executed dependently on the normality of the distribution. Receiver-operating characteristics (ROC) curve analysis was applied to confirm the best cut-off values of analyzed variables. Logistic regression analysis was used to determine odds ratios (ORs) with 95% confidence intervals (CIs). A *p*-value <0.05 was used to present statistical significance.

## RESULTS

### Patients' characteristics

Table 1 depicts the characteristics of the AOSD and sepsis patients. The median age was 36 (16-74) years for AOSD patients (male/female: 38/72) and 40 (18-71) years for sepsis patients (male/female: 37/52). Almost all the studied patients presented with fever (AOSD: 98.2%; sepsis: 98.8%). Besides, arthralgia/arthritis (52.7%), typical skin rash (44.5%), sore throat (35.5%), and myalgia (35.5%) were the main manifestations of AOSD other than sepsis. In addition, the laboratory characteristics of the entire AOSD and sepsis cohort has been presented in APPENDIX - Supplement Table 1.

The results showed no significant difference in the laboratory parameters between the test cohort and validation cohort in AOSD and sepsis patients (except WBC and neutrophil counts) (Table 2). This demonstrated that the results in the validation cohort could be used to confirm those in the test cohort.

### PMR and ferritin were independent factors in the test cohort

In the test cohort, the averages of PMR and serum ferritin levels among AOSD or sepsis patients were 26.87±12.19 and 7571.98±23723.03 μg/L or 18.03±10.21 and 565.06±568.92 μg/L, respectively (Table 2; *p<*0.001). CRP levels were intensively higher in the AOSD group than in the sepsis group (102.71±67.04 mg/L *vs* 69.81±56.70 mg/L, *p=*0.003). Furthermore, AOSD patients manifested with significantly elevated values of platelet and PCT but declined MPV values relative to sepsis patients (*p=*0.001; *p=*0.016; *p*<0.001). There was no obvious difference in sex and age, as well as values comprising WBC, lymphocyte, neutrophil, red cell distribution width (RDW), platelet distribution width (PDW), neutrophil to lymphocyte ratio (NLR), and PLT to lymphocyte ratio (PLR) between the two groups in the test cohort.

**Figure 1:**
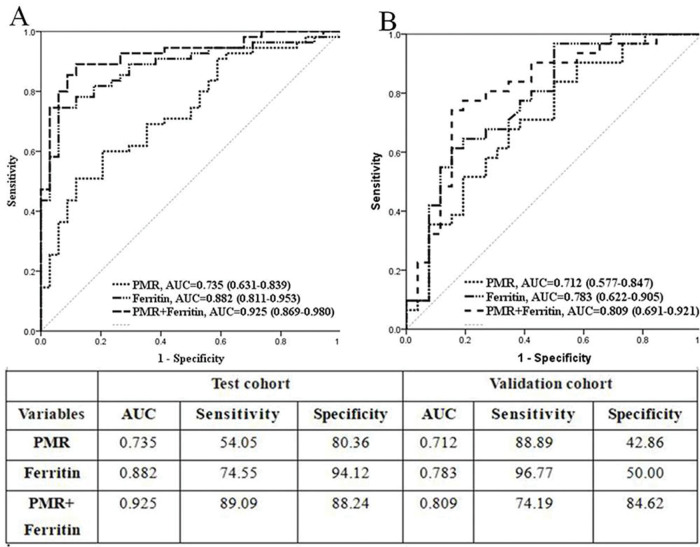
Receiver operating characteristic curve to compare the areas under the curve of PMR, ferritin, and the combination of PMR and ferritin in distinguishing AOSD from sepsis.

Next, ROC analysis was employed to recognize an optimal cutoff value for verification assessment; the value of PMR >25.06 (area under the ROC curve [AUC] 0.735, 95% CI 0.631-0.839, *p<*0.001) and ferritin level >1120 μg/L (AUC 0.882, 95% CI 0.811-0.953, *p<*0.001) were selected as potential predictors of AOSD. Then, the combined effectiveness of PMR and ferritin was also evaluated. The ROC analysis showed that the AUC of the combination showed an excellent value of 0.925 (95% CI 0.869-0.980, *p<*0.001) (Figure 1A). Hence, in the test cohort, we concluded that increased PMR values might be an independent indicator or a complementary indicator together with serum ferritin to indicate AOSD.

To identify the indicators that might help to distinguish AOSD from sepsis, potential factors, including ferritin, CRP, PLT, PCT, MPV, and PMR, were analyzed using the stepwise method by a multivariate binary logistic regression model. PMR and ferritin alone were distinct factors used to differentiate AOSD from sepsis (OR: 9.22, 95% CI 2.15-39.46, *p=*0.003; OR: 54.61, 95% CI 10.51-283.85, *p<*0.001; respectively) (Table 3). Moreover, PMR showed a significant positive link with serum CRP levels (r=0.253, *p=*0.004) and PLT (r=0.961, *p<*0.001) but an inverse correlation with RDW (r=-0.237, *p=*0.007), MPV (r=-0.607, *p<*0.001), PCT (r=0.838, *p<*0.001), and PDW (r=-0.403, *p<*0.001) (Table 4).

### ROC analysis in the validation cohort

We then verified the role of PMR, serum ferritin, and their combination in the validation cohort. The differential diagnostic value was evaluated in the validation cohort by AUC. The data showed that the AUC for PMR alone was 0.712 (95% CI 0.577-0.874, *p<*0.001) and that for ferritin was 0.783 (95% CI 0.662-0.905, *p<*0.001). The AUC for a combined model of PMR and ferritin was 0.809 (95% CI 0.691-0.927, *p<*0.001) (Figure 1B).

Thus, we confirmed that PMR is an independent risk factor that can identify AOSD from sepsis with a good diagnostic value. The identification efficiency was further elevated when combined with ferritin.

## DISCUSSION

AOSD is an uncommon inflammatory disease. Because of the lack of specific biomarkers, together with the fact that AOSD shares many clinical manifestations and serological indicators with other diseases, such as sepsis, misjudgments and delayed treatment is continuously prevailing. A study reported that antibiotics are initially used in 90% of AOSD patients with fever of unknown origin (12). It takes on average of four months from the disease onset to the final diagnosis of AOSD (13). Therefore, early diagnosis, and accurate and timely treatment can improve the prognosis of patients, and reduce pain and overutilization of medical resources (13,14). However, because of the lack of deep understanding of the mechanisms underlying the development of AOSD, the diagnostic efficiency has not been enhanced for a long time.

Previous literature reported that proinflammatory cytokines (interleukin [IL]-1, IL-6, and IL-18) were identified as key elements in AOSD (2). Certain clinical signs could help to differentiate AOSD from sepsis (15). Our results also confirmed that arthritis, rash, sore throat, and myalgia were more common in AOSD patients than in sepsis patients. However, there are individual differences in clinical signs, which may be ignored during a consultation. A specific and clinically useful serum biomarker is still lacking. Therefore, a useful and convenient marker is extremely required to compensate for symptom judgment to distinguish AOSD from sepsis.

Hematological parameters were proposed to be used as biomarkers for inflammatory diseases (16,17). MPV, as a part of platelet parameters, is an index of the PLT volume to reflect the PLT function state (18). MPV is associated with respiratory distress syndrome, necrotizing enterocolitis, bronchopulmonary dysplasia, and sepsis in newborns (19-21). Low levels of MPV can be used as an inflammation marker to reflect the severity of perianal abscesses, and high ferritin is associated with severe hyperinflammation in critically ill ICU patients (22,23). However, it is the first time that we seek to explore the role of MPR or its alternative form in AOSD and sepsis patients.

In this study, we comprehensively analyze the data of blood routine examination, serum CRP and ferritin. Univariate analysis results showed that ferritin, CRP, PLT, PCT, MPV, and the reciprocal form of MPR—PMR can be effective in distinguishing AOSD from sepsis. Multivariate analysis showed that PMR and ferritin alone are the independent effective indicators, thereby making it possible to assist physicians in differentiating AOSD from sepsis. In addition, ROC curve analysis confirmed that PMR and serum ferritin can identify AOSD from sepsis with a good diagnostic value in both the test cohort and validation cohort. Furthermore, PMR and serum ferritin are convenient and inexpensive laboratory serum markers. Thus, we speculated that PMR and ferritin could be used as biomarkers to distinguish AOSD from sepsis.

Given that a single indicator may not meet the demand in clinics, the combination of two or more indicators should be recommended to elevate predictive values (11,24). In our study, we also investigated if PMR combined with serum ferritin levels can distinguish AOSD from sepsis. The results exhibited that the AUC of the combination of PMR and serum ferritin was 0.925 (95% CI 0.869-0.980), which was superior to that of PMR or ferritin alone. In addition, the above results were further confirmed in the validation cohort. Therefore, PMR alone should be taken into consideration or combined with serum ferritin when clinicians have difficulty in differentiating AOSD from sepsis.

However, there are some limitations in the study: this was a single-center investigation with a small sample size; thus, a larger sample size is needed for further confirmation.

In conclusion, PMR can help distinguish AOSD from sepsis in the absence of interferences from other factors. PMR can be used as a sole indicator or a complementary indicator with serum ferritin to make a distinction between AOSD and sepsis.

## AUTHOR CONTRIBUTIONS

Zhou J designed the study. All of the authors contributed to the generation, collection, assembly, analysis, and/or interpretation of data. Ge S wrote the manuscript. Zhou J, Xie M, Qiao T and Ma Y revised the manuscript. All of the authors have read the manuscript and approved its final version.

## Figures and Tables

**Table 1:** Demographics and characteristics of the study participants.

Variables	Patients with AOSD (N=110)	Patients with sepsis (N=84)	*p*-value
Demographic variables			
Age, year	36 (16-74)	40 (16-71)	0.653
Female sex, n	72	52	0.612
Clinical features			
Fever, n	108 (98.2%)	83 (98.8%)	0.727
Arthralgia/arthritis, n	58 (52.7%)	16 (19.0%)	<0.001
Typical skin rash, n	49 (44.5%)	12 (14.3%)	<0.001
Sore throat, n	43 (35.5%)	11 (13.1%)	<0.001
Lymphadenopathy, n	14 (8.2%)	4 (4.8%)	0.059
Myalgia, n	37 (33.6%)	7 (8.3%)	<0.001
Hepatomegaly, n	2 (1.8%)	9 (10.7%)	0.008
Splenomegaly, n	5 (4.5%)	3 (3.6%)	0.737

AOSD: adult-onset Still’s disease.

**Table 2:** The laboratory characteristics of the patients included in the study.

			Pairwise comparison *p-*value					
	Test cohort		Validation cohort		Test *vs* Validation Cohort		Test cohort	Validation cohort
Variables	AOSD	Sepsis	AOSD	Sepsis	AOSD	Sepsis	AOSD *vs* Sepsis	
Sex (Male/Female)	26/47	24/32	12/25	13/15	0.854	0.759	0.407	0.258
Age	37.16±15.03	39.79±14.14	38.08±16.31	40.11±12.37	0.768	0.919	0.367	0.944
Ferritin	7571.98±23723.03	565.06±568.92	3604.77±3779.74	1467.80±2667.06	0.359	0.403	<0.001	<0.001
CRP	102.71±67.04	69.81±56.70	103.99±65.16	99.33±73.75	0.925	0.113	0.003	0.889
WBC	15.18±7.31	13.56±8.08	13.76±9.30	6.54±3.48	0.387	<0.001	0.253	<0.001
Lymphocyte	1.24±0.53	1.19±0.91	1.31±0.71	0.94±0.59	0.587	0.184	0.731	0.026
Neutrophil	13.35±7.23	11.65±7.57	11.76±9.11	5.06±3.27	0.323	<0.001	0.210	<0.001
RDW	13.97±1.86	13.84±1.47	14.25±2.07	14.34±2.56	0.480	0.261	0.847	0.958
PLT	252.74±107.31	187.87±94.64	261.47±121.40	76.54±119.19	0.702	0.637	0.001	0.004
PCT	0.24±0.11	0.20±0.09	0.26±0.11	0.18±0.11	0.467	0.383	0.016	0.003
MPV	9.69±1.31	10.85±1.24	10.22±1.13	10.43±1.17	0.039	0.146	<0.001	0.413
PDW	12.53±2.64	13.38±3.20	11.83±2.66	12.20±2.74	0.198	0.105	0.116	0.579
NLR	12.75±10.00	18.86±30.79	11.25±10.22	7.21±6.43	0.464	0.052	0.116	0.070
PLR	237.13±134.91	284.60±432.89	240.33±161.15	228.06±130.25	0.913	0.502	0.372	0.715
PMR	26.87±12.19	18.03±10.21	26.49±14.00	17.81±13.19	0.885	0.935	<0.001	0.007

AOSD: adult-onset Still’s disease; CRP: C-reactive protein; WBC: white blood cell; RDW: red cell distribution width; PLT: platelet; PCT: plateletcrit; MPV: mean platelet volume; PDW: platelet distribution width; NLR: neutrophil to lymphocyte ratio; PLR: PLT to lymphocyte ratio; PMR: PLT to MPV ratio.

**Table 3:** Univariate and multivariate analyses of factors for differentiating AOSD from sepsis.

	Univariate analysis			Multivariate analysis		
Variables	OR	95% CI	*p*-value	OR	95% CI	*p*-value
Ferritin	50.46	10.62-239.86	<0.001	54.61	10.51-283.85	<0.001
CRP	1.71	0.84-3.49	0.153			
PLT	2.73	1.33-5.61	0.008			
PCT	2.11	1.02-4.39	0.048			
MPV	3.86	1.84-8.06	<0.001			
PMR	2.89	1.41-5.96	0.004	9.22	2.15-39.46	0.003

AOSD: adult-onset Still’s disease; CRP: C-reactive protein; PLT: platelet; PCT: plateletcrit; MPV: mean platelet volume; PDW: platelet distribution width; PMR: PLT to MPV ratio**.**

**Table 4:** Correlation between PLT to MPV ratio and variables.

Variables	Correlation coefficient (r)	*p*-value
Ferritin (μg/L)	-0.124	0.250
CRP (mg/L)	0.253	0.004
WBC (×10^9^/L)	0.169	0.055
Lymphocyte (×10^9^/L)	0.098	0.270
Neutrophil (×10^9^/L)	0.164	0.063
RDW	-0.237	0.007
PLT (×10^9^/L)	0.961	<0.001
PCT (%)	0.838	<0.001
MPV (fL)	-0.607	<0.001
PDW (%)	-0.403	<0.001

CRP: C-reactive protein; WBC: white blood cell; RDW: red cell distribution width; PLT: platelet; PCT: plateletcrit; MPV: mean platelet volume; PDW: platelet distribution width.

## APPENDIX

**Supplementary Table:** Clinical data of AOSD and sepsis patients.

Variables	Patients with AOSD	Patients with sepsis	*p*-value
Ferritin	6141.94±19137.53	956.25±1843.53	<0.001
CRP	103.13±66.12	78.63±63.25	0.012
WBC	14.71±8.00	11.22±7.63	0.002
Lymphocyte	1.26±0.59	1.11±0.82	0.130
Neutrophil	12.83±7.89	9.45±7.15	0.002
RDW	14.06±1.93	14.01±1.90	0.858
PLT	255.60±111.63	184.10±102.89	<0.001
PCT	0.25±0.11	0.19±0.10	<0.001
MPV	9.86±1.27	10.71±1.23	<0.001
PDW	12.30±2.65	12.98±3.09	0.102
NLR	9.8±1.2	14.98±12.93	0.070
PLR	238.18±142.79	265.76±361.13	0.466
PMR	26.75±12.75	17.95±11.21	<0.001
PPR	22.39±15.74	15.74±10.70	<0.001
